# Factors Associated with Early Mortality in HIV-Positive Men and Women Investigated for Tuberculosis at Ethiopian Health Centers

**DOI:** 10.1371/journal.pone.0156602

**Published:** 2016-06-07

**Authors:** Anton Reepalu, Taye Tolera Balcha, Sten Skogmar, Nuray Güner, Erik Sturegård, Per Björkman

**Affiliations:** 1 Infectious Diseases Research Unit, Department of Clinical Sciences, Faculty of Medicine, Lund University, Malmö, Sweden; 2 Ministry of Health, Addis Ababa, Ethiopia; 3 Region Skåne Competence Center, Skåne University Hospital, Lund, Sweden; 4 Medical Microbiology, Department of Laboratory Medicine, Malmö, Lund University, Lund, Sweden; FIOCRUZ, BRAZIL

## Abstract

**Introduction:**

Despite increasing access to antiretroviral treatment (ART) in low-income countries, HIV-related mortality is high, especially in the first months following ART initiation. We aimed to evaluate the impact of TB coinfection on early mortality and to assess gender-specific predictors of mortality in a cohort of Ethiopian adults subjected to intensified casefinding for active TB before starting ART.

**Material and Methods:**

Prospectively recruited ART-eligible adults (n = 812, 58.6% female) at five Ethiopian health centers were followed for 6 months. At inclusion sputum culture, Xpert MTB/RIF, and smear microscopy were performed (158/812 [19.5%] had TB). Primary outcome was all-cause mortality. We used multivariate Cox models to identify predictors of mortality.

**Results:**

In total, 37/812 (4.6%) participants died, 12 (32.4%) of whom had TB. Karnofsky performance score (KPS) and mid-upper arm circumference (MUAC) were associated with mortality in the whole population. However, the associations were different in men and women. In men, only MUAC remained associated with mortality (adjusted hazard ratio [aHR] 0.71 [95% CI 0.57–0.88]). In women, KPS <80% was associated with mortality (aHR 10.95 [95% CI 2.33–51.49]), as well as presence of cough (aHR 3.98 [95% CI 1.10–14.36]). Cough was also associated with mortality for TB cases (aHR 8.30 [95% CI 1.06–65.14]), but not for non-TB cases.

**Conclusions:**

In HIV-positive Ethiopian adults managed at health centers, mortality was associated with reduced performance score and malnutrition, with different distribution with regard to gender and TB coinfection. These robust variables could be used at clinic registration to identify persons at increased risk of early mortality.

## Introduction

Although survival in people living with HIV (PLWH) has increased during the last decade as a result of expanding access to antiretroviral treatment (ART), HIV-related mortality remains high, especially in sub-Saharan Africa where 800,000 deaths were caused by HIV/AIDS in 2014 [1]. The risk of death among PLWH mainly depends on the presence of coinfections and the severity of immunosuppression at ART initiation [2–4]. Since clinical and immunological outcomes are better if ART is started before advanced immunosuppression has developed [5,6], current WHO guidelines recommend that ART should be initiated in everyone living with HIV irrespective of CD4 cell count [7].

Despite these revised criteria for ART, large proportions of PLWH still present with advanced immunosuppression at HIV diagnosis. According to a recent meta-analysis estimating temporal trends in disease status at presentation to care and ART initiation, mean CD4 cell counts did not increase significantly from 2002 to 2012 in sub-Saharan Africa [8]. Although all PLWH have an indication to start ART, subjects with advanced immunosuppression must be identified and prioritized for early ART initiation due to their elevated risk of death [7]. This is illustrated by studies from South Africa that show great accumulated mortality with delayed treatment initiation during the first month after registration for patients with CD4 cell counts <50 cells/μL and/or WHO stage IV disease [4,9].

Besides CD4 cell count at ART initiation, other factors may be involved in HIV-related mortality. For example, low body-mass index (BMI), anaemia, and increasing age have been associated with elevated risk of death in PLWH starting ART [2,10,11]. Risk factors for death in HIV infection may also vary with regard to gender. Male PLWH are often reported to have excess mortality after starting ART compared to female PLWH [10], but gender-specific risk factors for mortality have not been thoroughly studied [12].

Importantly, the underlying causes of death in PLWH show regional variations, and are incompletely understood. In sub-Saharan Africa, most fatalities in PLWH are considered to be caused by tuberculosis (TB) [13]. This is supported by autopsy studies [14–16], revealing high rates of TB, also in persons who had been investigated for TB before death [3]. Although correct diagnosis and treatment of TB is recognized as a critical component of HIV care [17], identification of TB in PLWH is difficult, and it is likely that active TB is missed in many persons starting ART in resource-limited settings.

We have previously presented data on TB coinfection and ART outcomes in a cohort of treatment-naïve HIV-positive adults who met criteria for ART recruited at Ethiopian health centers [18]. Despite intensified casefinding for TB and access to therapy for both TB and HIV around 5% of participants died during the first 6 months after inclusion. We hypothesized that robust clinical characteristics present at inclusion, might be used to identify individuals at high risk of 6-month mortality in routine health care. Since both TB coinfection and male gender have been associated with excess mortality in other studies, we determined predictors of early mortality in this cohort specifically for men and women as well as for participants with and without TB coinfection. Furthermore, considering that most patients who present to HIV care do not initiate ART directly at presentation, we analyzed mortality from the time of first clinic visit instead of from the time of ART initiation.

## Material

The cohort used for this study was recruited to determine the prevalence of active TB in ART eligible patients at Ethiopian health centers, to evaluate different diagnostic methods for TB and to determine the outcome of ART and TB treatment. The cohort and study setting have previously been described in detail [18,19]. In brief, PLWH presenting at all five public health centers providing HIV care in the city of Adama, Oromia region, central Ethiopia, and adjacent rural and suburban disctricts were assessed for eligibility. The health centers (2 urban, 2 semi-urban, 1 rural) are situated on the highway connecting Addis Ababa and Djibouti, which is considered to be a high-risk corridor for HIV infection in Ethiopia. Non-physician clinicians (nurses or health officers with 3–4 years of academic training) deliver all care at these health centers. Inclusion criteria for the cohort were: age 18 years or greater, indication for ART according to Ethiopian guidelines at the time of recruitment (CD4 cell count <350 cells/μL and/or WHO stage IV), and residency within the catchment area. Patients with previous or current ART and/or TB treatment for more than 2 weeks at the time of screening were excluded. Participants were recruited from October 2011 until March 2013. The cohort is continuously followed with regular study visits to evaluate the long-term outcome of ART.

## Methods

At inclusion, trained non-physician clinicians collected detailed clinical data, including findings from physical examination, following a structured questionnaire. All participants (irrespective of symptoms) were requested to provide sputum samples for TB diagnostics by liquid culture, GeneXpert MTB/RIF, and smear microscopy. In case of peripheral lymphadenopathy, TB diagnostics were performed on fine-needle aspirates. Patients who did not submit sputum samples were excluded from the cohort since they could not be categorized for TB. Blood samples were obtained for complete blood and CD4 cell counts. Results from blood tests and TB investigations were available to the responsible clinicians. Detailed description of the TB diagnostic procedure has been published previously [19]. For this study a participant was defined as a TB case either by bacteriological confirmation (positive result by any of the three bacteriological methods) or clinical criteria. Cases of clinically diagnosed TB fulfilled criteria according to the Ethiopian National TB guidelines (symptoms and/or signs compatible with active TB supported by radiological results and lack of response to antibiotic therapy) [20]. All TB cases diagnosed within 3 months after inclusion were considered as prevalent TB.

Follow-up visits were scheduled at months 1, 2, 3, and 6 after study inclusion. In case of symptoms or signs suggestive of TB during follow-up study clinicians were encouraged to repeat TB diagnostics according to the baseline protocol. Health extension workers traced patients with missed appointments by telephone and home visits. The health center clinicians were responsible for starting ART and treating opportunistic infections (including TB) in accordance with national guidelines [20,21], without the involvement of the study investigators. Study data was continuously entered into an electronic database with regular crosschecking by trained data clerks.

### Statistical analysis

The primary outcome for this study was all-cause mortality within 6 months of study inclusion. Analysis of predictors for this outcome was based on characteristics collected at study inclusion. This analysis was first performed for the whole population, and then separately with regard to gender and TB status, respectively. Time-at-risk was defined as days from inclusion until time of death or 6 months after inclusion. Patients more than 3 months late for a scheduled visit were defined as lost to follow-up (LTFU) and their follow-up time was right censored at the last study visit. For patients who transferred their care to other facilities or who declined further follow-up similar right censoring was used.

Patient characteristics were summarized using frequencies and percentages for categorical variables and median and interquartile range (IQR) for continuous variables. For all continuous variables, plots of beta estimates for the primary outcome for each quintile of the variable were assessed to identify each variable’s functional form in order to define appropriate cut-off values. Mid-upper arm circumference (MUAC) and BMI were kept as continuous variables. CD4 cell count was categorized by quartiles, and haemoglobin according to the WHO anaemia cut-offs, merging no anaemia (haemoglobin ≥13.0 and ≥12.0 g/dL for men and women, respectively) and mild anaemia (haemoglobin 11.0–12.9 g/dL and 11.0–11.9 g/dL for men and women, respectively). Karnofsky performance score (KPS) was dichotomized at <80% differentiating those unable to carry out normal activities or do active work. Patient characteristics for male and female participants, as well as for TB cases and non-TB cases, were compared using χ^2^-test for categorical, and Mann-Whitney’s U-test for continuous variables.

Kaplan-Meier survival analyses were used to investigate overall survival from inclusion, using the log rank test to compare male and female participants as well as TB cases and non-TB cases, respectively. Cox proportional hazards models were used to investigate baseline variables’ correlation with mortality. In order to further assess associations with mortality, four separate models were fitted for males, females, TB cases, and non-TB cases, respectively. All variables considered for the Cox models were assessed for the proportional hazards assumption using Kaplan-Meier and log minus log plots, and by analysing each variables’ interaction with time. Only variables fulfilling the assumption were included in the final models.

Variables from each univariate model with a p value <0.3 were entered into multivariate models, whereby the least significant variables were removed using a backward stepwise procedure until only variables with p<0.05 remained in the model. At each step, the beta estimates of variables remaining in the model were analysed for possible interaction with the variable removed. A change in beta estimate of >20% was considered to indicate a possible interaction, which then was further evaluated. The multivariate models were adjusted for age and CD4 cell count, as well as ART status, included as a time-varying 0/1 variable. Time-to-ART was defined as days from study inclusion until start of ART.

To account for missing data, secondary analyses were performed in which the multivariate models were refitted excluding variables with <95% available data from the start of the stepwise procedure. The final model generated was then compared with the original model to assess the possible bias derived from cases with missing data.

For all analyses, a p value <0.05 was considered to indicate statistical significance. All analyses were performed using SPSS, version 21 (IBM Corp, Armonk, NY).

### Ethical approval

Ethical approval was obtained from the National Research Ethics Committee at the Ministry of Science and Technology of Ethiopia and the Regional Ethical Review Board of Lund University, Sweden. All study participants provided written informed consent.

The health center clinicians were responsible for all aspects of HIV and TB care in line with national guidelines. All laboratory results were communicated back to the responsible clinician, including all TB results. No treatments were withheld from the patients due to this study.

## Results

### Participants

**Fig 1:**
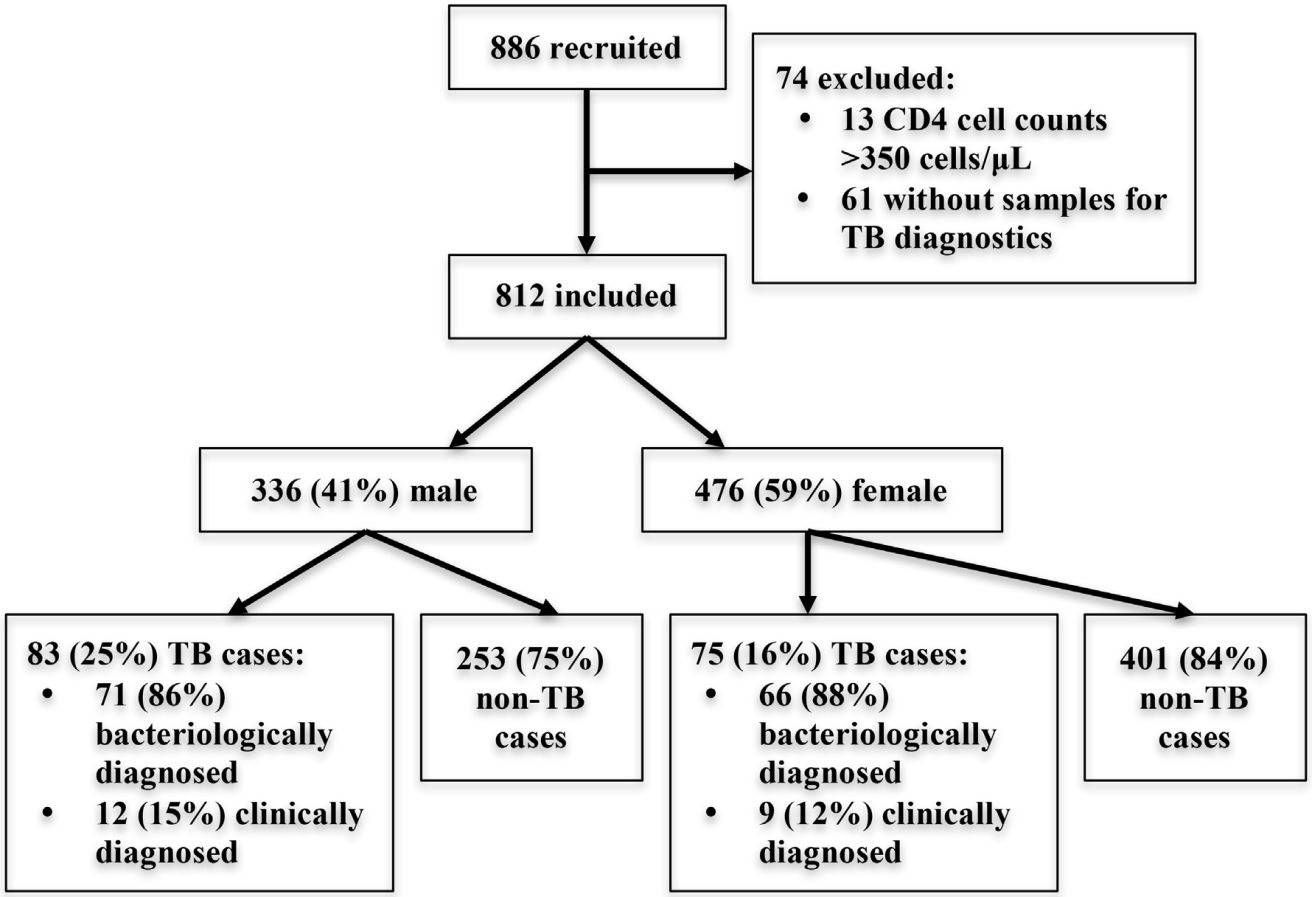
Flow chart of study participants.

A total of 886 PLWH were screened for eligibility; 74 were excluded and 812 participants (58.6% female) were included in the study cohort (Fig 1). Among these, 158 (19.5%) were diagnosed with TB (137 [86.7%] bacteriologically confirmed, 21 [13.3%] clinically diagnosed, Fig 1). Characteristics of study participants are shown in Table 1. The median age of men was higher than that of women (p<0.01). Median CD4 cell counts were lower in men compared to women (p = 0.01), and in subjects with compared to those without TB (p<0.01). No case of incident TB was diagnosed during the 6-month follow-up period.

**Table 1:** Characteristics of study participants at inclusion.

	All n = 812	Male n = 336	Female n = 476	*P*^1^	TB cases n = 158	Non-TB cases n = 654	*P*^2^
**Age—years**	32 (28–40)	35 (30–42)	30 (26–36)	**<0.01**	34 (28–40)	32 (28–39)	0.18
**Gender—female**	476 (59)	–	–		75 (47)	401 (61)	**<0.01**
**BMI—kg/m**^**2**^	18.9 (17.3–21.0)	18.8 (17.2–20.3)	19.1 (17.4–21.6)	**<0.01**	17.7 (16.3–19.6)	19.2 (17.6–21.3)	**<0.01**
**MUAC—cm**	23 (21–24)	23 (21–24)	22 (20–24)	0.51	21 (19–23)	23 (21–25)	**<0.01**
**CD4 cell count—cells/μL**	208 (116–320)	191 (104–302)	219 (132–331)	**0.01**	170 (91–273)	220 (127–328)	**<0.01**
**>300**	229 (28)	85 (26)	144 (30)		33 (21)	196 (30)	
**201–300**	197 (24)	76 (23)	121 (26)		27 (17)	170 (26)	
**100–200**	223 (28)	93 (28)	130 (27)		53 (34)	170 (26)	
**<100**	158 (20)	78 (24)	80 (17)		45 (28)	113 (17)	
**CD4 cell count—%-age**	12 (8–17)	11 (7–16)	13 (9–17)	**<0.01**	10 (9–12)	12 (8–17)	0.41
**TB coinfection**	158 (19)	83 (25)	75 (16)	**<0.01**	–	–	
**Haemoglobin, g/dL**				0.11			**<0.01**
**>10.9**	467 (61)	206 (66)	261 (58)		56 (38)	411 (67)	
**8.0–10.9**	268 (35)	97 (31)	171 (38)		79 (53)	189 (31)	
**<8.0**	27 (4)	11 (4)	16 (4)		14 (9)	13 (2)	
**In care at study inclusion**	557 (69)	218 (65)	339 (72)	0.05	93 (59)	464 (71)	**<0.01**
**HIV test due to symptoms**	442 (55)	205 (61)	237 (50)	**<0.01**	107 (68)	335 (51)	**<0.01**
**Signs and symptoms**							
**Weight loss**	514 (64)	238 (71)	276 (58)	**<0.01**	130 (82)	384 (59)	**<0.01**
**Appetite loss**	401 (50)	168 (50)	233 (49)	0.71	101 (64)	300 (46)	**<0.01**
**Fatigue**	553 (69)	233 (71)	320 (69)	0.56	131 (85)	422 (66)	**<0.01**
**Fever**	389 (48)	165 (49)	224 (47)	0.58	100 (64)	289 (44)	**<0.01**
**Cough**	326 (40)	149 (44)	177 (37)	**0.04**	98 (62)	228 (35)	**<0.01**
**Night sweats**	396 (49)	174 (52)	222 (47)	0.16	113 (72)	283 (43)	**<0.01**
**Conjunctive pallor**	126 (16)	58 (18)	68 (14)	0.22	41 (26)	85 (13)	**<0.01**
**Karnofsky score <80%**	275 (34)	120 (36)	155 (33)	0.35	89 (56)	186 (28)	**<0.01**
**WHO clinical stage**				**0.01**			**<0.01**
**Stage 1**	141 (17)	54 (16)	87 (18)		13 (8)	128 (20)	
**Stage 2**	246 (30)	84 (25)	162 (34)		31 (20)	215 (33)	
**Stage 3**	322 (40)	146 (44)	176 (37)		76 (48)	246 (38)	
**Stage 4**	100 (12)	50 (15)	50 (11)		28 (24)	62 (10)	

Data presented as n (%), or median (interquartile range). Data available for >97% for all variables, except haemoglobin with 762/812 (93.8%) available data.

Abbreviations: BMI, body-mass index; MUAC, mid-upper arm circumference; TB, tuberculosis.

^1^*P* value comparing male and female using χ^2^ test for categorical and Mann-Whitney’s U-test for continuous variables.

^2^*P* value comparing TB cases and non-TB cases using χ^2^ test for categorical and Mann-Whitney’s U-test for continuous variables.

### Survival and retention in care

At 6 months after inclusion 679 participants (83.6%) remained in care, 37 (4.6%) were confirmed dead, 42 (5.2%) LTFU, 35 (4.3%) had registered transfer of care, and 20 (2.5%) declined further follow-up. ART was started in 564 (69.5%) subjects a median of 27 days (IQR 12–57) after inclusion. The median time to death for the 37 deceased participants was 54 days (IQR 30–87); 18 (48.6%) died before and 19 (51.4%) died after starting ART. In the latter group, death occurred a median of 51 days (IQR 33–72) after starting ART. In total, 12 (32.4%) of the deceased participants had been diagnosed with TB; 8 (66.7%) of these had not started ART and 4 (33.3%) died after having started ART (Table 2). Among the 25 deceased non-TB cases 10 (40.0%) had not started ART and 15 (60.0%) died after having started ART. The median age of deceased TB cases was 30 years (IQR 28–35; 5 male, 7 female), as compared to 35 years (IQR 28–42; 15 male, 10 female) for non-TB cases.

**Table 2:** Mortality distribution with regard to ART, gender and TB status.

	Mortality		
	Total	Before ART start	After ART start
**All**	37/812 (4.6)	18 (48.6)	19 (51.4)
By gender:			
**Male**	20/336 (6.0)	9 (45.0)	11 (55.0)
**Female**	17/476 (3.6)	9 (52.9)	8 (47.1)
By TB status:			
**TB-cases**^1^	12/158 (7.6)	8 (66.7)	4 (33.3)
**Non-TB cases**	25/654 (3.8)	10 (40.0)	15 (60.0)

Presented as n/N (%) and n (%). Abbreviations: ART, antiretroviral treatment.

^1^6 (50.0%) of TB cases died before starting TB treatment.

**Fig 2:**
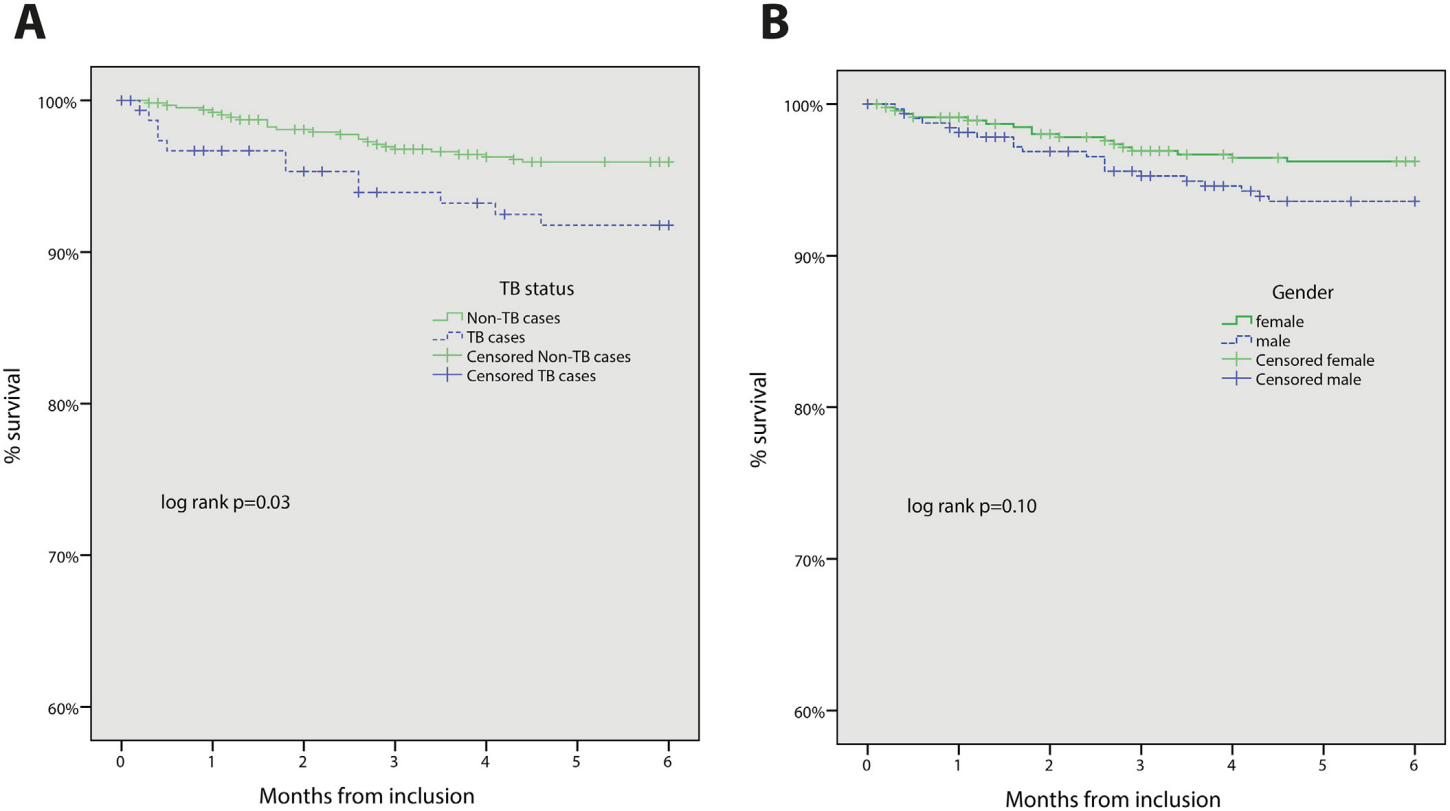
Kaplan-Meier plots comparing survival by gender (A) and tuberculosis coinfection (B).

No significant difference was found when comparing survival by gender (p = 0.10) using Kaplan-Meier plots. Non-TB cases had significantly better survival than TB cases (p = 0.03, Fig 2).

### Factors associated with mortality

Complete univariate hazard ratios for all tested variables are presented in S1 Table.

In the multivariate analysis for the entire cohort, KPS<80% and MUAC were independently associated with mortality (Table 3). The adjusted hazard ratio (aHR) for KPS<80% was 4.30 (95% CI 1.84–10.08). For each centimeter increase in MUAC the risk of mortality decreased with an aHR 0.82 (95% CI 0.71–0.94).

**Table 3:** Final multivariate Cox models for mortality after stepwise removal of non-significant variables; five separate models for all, males, females, TB cases and non-TB cases.

Subgroup and variables	aHR (95% CI)
**All**	
Karnofsky score <80%	4.30 (1.84–10.08)
MUAC—per cm increase	0.82 (0.71–0.94)
**Male**	
MUAC—per cm increase	0.71 (0.57–0.88)
CD4 cell count—cells/μL	
>300	1.00
201–300	1.82 (0.25–13.23)
100–200	1.95 (0.34–11.18)
<100	6.80 (1.37–33.78)
**Female**	
Karnofsky score <80%	10.95 (2.33–51.49)
Reported cough	3.98 (1.10–14.36)
**TB cases**	
Reported cough	8.30 (1.06–65.14)
**Non-TB cases**	
Gender, male vs. female	2.53 (1.05–6.12)
Karnofsky score <80%	4.89 (1.81–13.19)
MUAC—per cm increase	0.76 (0.63–0.91)

Abbreviations: aHR, adjusted hazard ratio; CI, confidence interval; MUAC, mid-upper arm circumference.

Adjusted for age as a continuous variable, CD4 cell counts as a categorical variable and antiretroviral treatment as a time-varying variable.

Among men MUAC had a stronger negative association with mortality, aHR 0.71 (95% CI 0.57–0.88); however, KPS<80 was not associated with mortality in men. Furthermore, male participants with CD4 cell counts <100 cells/μL had an aHR of 6.80 (95% CI 1.37–33.78) compared with CD4 cell counts >300 cells/μL.

For women KPS<80% had the overall strongest correlation with mortality with aHR 10.95 (95% CI 2.33–51.49), but the association between MUAC and mortality did not reach statistical significance. Reported cough at study inclusion was independently associated with an increased risk of early mortality for female participants, aHR 3.98 (1.10–14.36).

In the multivariate model for TB cases, reported cough was the only variable that remained in the final model, aHR 8.30 (1.06–65.14).

Among participants not diagnosed with TB male gender was associated with mortality (aHR 2.53 [95% CI 1.05–6.12]), as well as KPS<80% aHR 4.89 (1.81–13.19) and MUAC aHR 0.76 (95% CI 0.63–0.91) per centimeter increase (Table 3).

Haemoglobin was missing for 50 (6.2%) participants. Therefore, all multivariate models were refitted excluding anaemia from the start. The final models did not change.

## Discussion

In this cohort of HIV-positive Ethiopian adults, mortality during the first 6 months after inclusion was associated with reduced performance score and malnutrition (assessed by MUAC measurement). As part of the cohort study protocol, all participants had been investigated for active TB at inclusion. Although this resulted in detection of TB in 19.5% of subjects, persons with TB were at increased risk of death.

In contrast to other studies [2,10,11,22], we did not observe gender-related differences in the risk of death. It is possible that we did not observe a statistically significant survival difference for men versus women due to insufficient statistical power. However, we did find that factors associated with early mortality showed clear variations depending on gender. Thus, the significant association with lower MUAC was restricted to men, and low KPS to women. Interestingly, CD4 cell count—considered the most important predictor of HIV progression—was independently associated with mortality only in the subset of men with levels <100 cells/μL, as compared to men with >300 cells/μL. Among reported symptoms, cough was associated with death in women. This symptom was also the only variable that showed independent association with death among TB-cases.

In this study, time-at-risk started at the time of study inclusion and we did not restrict the analysis of mortality to those who actually started ART. This design allowed us to estimate the proportion and risk factors for death also in ART-eligible persons who died before starting treatment. The median time between inclusion and death for deceased subjects was short (48 days), and they had advanced disease at presentation. In particular, a greater proportion had TB [18,19].

In the current WHO guidelines all PLWH should start ART [7]. However, HIV/TB coinfected patients should start antituberculosis treatment before ART. Coinfected patients with profound immunosuppression should receive ART immediately within the first 2 weeks of TB treatment. Otherwise ART should be started as soon as possible within the first 8 weeks of TB treatment [23]. These recommendations are based on three key clinical trials that showed a significantly improved survival for coinfected patients if ART was started during the course of TB treatment [24–26]. Ethiopian guidelines for ART are concordant with the WHO recommendations [20]. It is possible that earlier consideration of ART for the study participants in our cohort that died could have improved survival, although our study was not designed to determine this.

In this study we aimed to include potential markers of mortality risk that would be possible to use at health center level in low-income countries. Based on previous findings from our own studies and on the scientific literature, we hypothesized that poor performance status and malnutrition would be associated with mortality. Karnofsky performance score was chosen as we wanted a general, standardized, and established measure of the performance status of participants that could be easily recorded by health care workers. Malnutrition can be measured using BMI and/or MUAC. Both our research group and others have shown independent association between MUAC and mortality in patients with TB [27,28]. Furthermore, we have also shown that low MUAC is associated with prevalent TB in HIV-positive adults [29]. A benefit of MUAC over BMI is the minimal need of equipment; all that is needed is a measuring tape. In line with our hypotheses, we found that low performance status and MUAC were independently associated with the risk of early death, whereas the level of CD4 cells did not have capacity to predict mortality. This suggests that these simple measurements might be used at HIV clinics to identify high-risk individuals. What interventions should then be undertaken to reduce the risk of death in patients manifesting these “warning signs”? Such persons should be prioritized for early ART initiation, which leads to improved survival [4,9]. Yet, half of patients who died had started ART. In agreement with another cohort study from a resource-limited setting most of these deaths occurred within the first 3 months after starting ART (in median 51 days after ART) [4].

Unfortunately, causes of death were not determined for our participants. This reflects the usual situation in low-income countries, and highlights the importance of detailed post-mortem investigation studies. We could ascertain, primarily by telephone calls to the family of the deceased, that the deaths in our study were due to illness and not attributable to accidents or other unnatural causes. It is possible that use of verbal autopsies could have elucidated causes of death with some greater accuracy [30]. In autopsy studies from sub-Saharan Africa TB has been reported as a leading cause of death; Wong et al found mycobacterial infections to be implicated in 69% of deaths in a cohort of HIV-positive adults in Johannesburg, South Africa [15], and Bates et al reported TB in 66/101 (65%) HIV-positive patients autopsied in Lusaka, Zambia [16].

In our cohort, all participants had undergone active TB casefinding at inclusion, leading to diagnosis of TB in 158/812 persons, which in most cases (91.8%) had not been identified previously. Among participants not diagnosed with TB in our cohort, mortality was associated with male gender, low KPS and MUAC. Interestingly, these latter variables have also been found to be independently associated with prevalent TB in this cohort [29]. Self-reported cough was independently associated with death in women but not in men; in fact, among the non-TB cases that died 80.0% of women reported cough compared with 33.3% of the men. This phenomenon may be related to higher rates of poor quality sputum samples in women than men [31], and could indicate missed cases of pulmonary TB in women. Since our diagnostic protocol was focused on pulmonary TB it is possible that some cases of TB, in particular extrapulmonary disease, may have been missed, suggesting that the real burden of active TB in this population may be even higher than that found through sputum testing. Because of the difficulty in diagnosing TB in PLWH, and the severe consequences of missed TB in this population, some trials have evaluated the effect of empiric TB treatment to PLWH with advanced immunosuppression. Currently, the role of this treatment strategy is not obvious; in the REMEMBER trial empiric TB treatment did not lead to decreased mortality compared to standard care [32]. Using predictors for early mortality, such as those presented here might help to identify individuals who could benefit from empiric TB treatment [33].

This study was based on a large material of patients with structured and detailed categorization for active TB at inclusion. Furthermore, participants were recruited at Ethiopian public health centers, representing a typical health care setting for where most PLWH receive care in low-income countries.

Our study has certain limitations. For a considerable proportion of patients the outcome at 6 months was unknown (LTFU 42/812; 5.2%). LTFU is common in ART programs in sub-Saharan Africa, and studies on LTFU have identified different reasons for this phenomenon [34–36]. In a study from Ethiopia around 50% of subjects LTFU from an ART clinic were found to be dead when tracing was done [36]; other explanations may be “self-transfer” of care and resort to alternative therapies. In contrast to patients with confirmed death, characteristics of participants LTFU did not differ significantly from those remaining in care. However, their characteristics did differ from those that died. Median CD4 cell count was 99 cells/mm^3^ and 198 cells/mm^3^, median MUAC was 20 cm and 22 cm, and TB prevalence was 32% and 17%, for patients that died and were LTFU, respectively (S1 File). Our study did not aim to investigate reasons for LTFU. As part of our study protocol, we attempted to trace patients that were lost, but for a proportion this tracing was not successful. Although we could not determine the proportion of cases LTFU that have died, we do not consider early morality to be a major outcome in this group. That 5.2% in our study were lost within 6 months is a major limitation. However, for the reasons mentioned above we do not consider the proportion of participants categorized as LTFU as having had any significant impact on the outcome of the study. Furthermore, we excluded persons who did not provide sputum samples for TB testing at inclusion, and TB investigations were mainly restricted to pulmonary disease. Before implementing the use of the predictors identified here in routine care it will be necessary to validate our findings in external cohorts.

## Conclusions

In conclusion, we found that two simple clinical measurements have strong independent association with 6-month mortality in Ethiopian PLWH eligible to start ART: MUAC for men, and KPS for women. These variables could be considered for use in routine care to identify subjects at high risk of early mortality. Such persons may particularly benefit from fast-track ART initiation, as well as intensified investigation for TB.

## Supporting Information

S1 File The raw data underlying all statistical analyses in the manuscript. (SAV)

S1 Table Univariate Cox proportional hazards models for eraly mortality, separate models for all, male, female, TB cases, and non-TB cases, respectively. (DOCX)
