# Supplementary material for: Factors Associated with Early Mortality in HIV-Positive Men and Women Investigated for Tuberculosis at Ethiopian Health Centers
Source: PLoS One. 2016 Jun 7;11(6):e0156602. doi: 10.1371/journal.pone.0156602 (PMC4896420; doi:10.1371/journal.pone.0156602)
Supplement: S1 Table — (DOCX) [file pone.0156602.s002.docx]

**S1 Table. Univariate Cox proportional hazards models for early mortality, separate models for all, male, female, TB cases, and non-TB cases, respectively.**

|  | All | | Male | | Female | | TB cases | | Non-TB cases | |
| --- | --- | --- | --- | --- | --- | --- | --- | --- | --- | --- |
| Variable | **HR (95% CI)** | **p** | **HR (95% CI)** | **p** | **HR (95% CI)** | **p** | **HR (95% CI)** | **p** | **HR (95% CI)** | **p** |
| Age – per year | 1.00 (0.96-1.03) | 0.902 | 1.00 (0.95.1.05) | 0.907 | 0.98 (0.92-1.04) | 0.470 | 0.94 (0.88-1.02) | **0.125** | 1.02 (0.98-1.06) | 0.427 |
| Gender – female vs. male | 1.71 (0.90-3.27) | **0.102** | – |  | – |  | 0.61 (0.20-1.93) | 0.403 | 2.48 (1.12-5.53) | **0.026** |
| BMI – per 1 kg/m^2^ | 0.77 (0.67-0.88) | **<0.001** | 0.70 (0.58-0.84) | **<0.001** | 0.84 (0.70-1.01) | **0.057** | 0.93 (0.74-1.18) | 0.541 | 0.71 (0.59-0.85) | **<0.001** |
| MUAC – per cm | 0.72 (0.63-0.82) | **<0.001** | 0.68 (0.57-0.83) | **<0.001** | 0.73 (0.60-0.87) | **0.001** | 0.77 (0.60-0.99) | **0.043** | 0.71 (0.61-0.83) | **<0.001** |
| CD4 cell count – cells/µL |  |  |  |  |  |  |  |  |  |  |
| >300 | 1.0 |  | 1.0 |  | 1.0 |  | 1.0 |  | 1.0 |  |
| 201-300 | 1.00 (0.31-3.28) | 0.999 | 1.16 (0.16-8.24) | 0.881 | 0.92 (0.21-4.10) | 0.911 | 4.05 (0.42-38.92) | 0.226 | 0.47 (0.09-2.43) | 0.368 |
| 100-200 | 1.26 (0.42-3.75) | 0.678 | 1.93 (0.35-10.53) | 0.449 | 0.87 (0.20-3.90) | 0.859 | 0.65 (0.04-10.32) | 0.757 | 1.46 (0.45-4.77) | 0.534 |
| <100 | 4.82 (1.91-12.15) | **0.001** | 6.68 (1.48-30.14) | **0.014** | 3.47 (1.02-11.87) | **0.047** | 6.14 (0.76-49.94) | **0.090** | 4.10 (1.43-11.81) | **0.009** |
| CD4 cell %-age – per % | 0.97 (0.92-1.02) | **0.245** | 0.96 (0.89-1.03) | **0.283** | 0.99 (0.92-1.07) | 0.792 | 1.01 (0.94-1.09) | 0.851 | 0.95 (0.89-1.01) | **0.124** |
| TB coinfection | 2.09 (1.05-4.16) | **0.036** | 1.01 (0.37-2.78) | **0.985** | 4.14 (1.57-10.87) | **0.004** | – |  | – |  |
| Haemoglobin, g/dL |  |  |  |  |  |  |  |  |  |  |
| >10.9 | 1.0 |  | 1.0 |  | 1.0 |  | 1.0 |  | 1.0 |  |
| 8.0-10.9 | 1.97 (0.92-4.18) | **0.079** | 2.52 (0.92-6.96) | **0.074** | 1.61 (0.52-4.99) | 0.409 | 0.77 (0.19-3.09) | 0.716 | 2.49 (1.01-6.12) | **0.047** |
| <8.0 | 7.64 (2.72-21.44) | **<0.001** | 6.00 (1.25-28.90) | **0.025** | 9.48 (2.37-37.91) | **0.001** | 3.47 (0.78-15.50) | **0.104** | 8.05 (1-74-37.26) | **0.008** |
| In HIV care at inclusion | 0.41 (0.22-0.79) | **0.007** | 0.54 (0.22-1.29) | **0.165** | 0.33 (0.13-0.87) | **0.024** | 0.46 (0.15-1.46) | **0.189** | 0.43 (0.20-0.94) | **0.035** |
| HIV test due to symptoms | 1.83 (0.92-3.64) | **0.085** | 1.56 (0.60-4.05) | 0.364 | 1.94 (0.72-5.24) | **0.193** | 0.96 (0.29-3.19) | 0.945 | 2.13 (0.92-4.94) | **0.078** |
| Signs and symptoms |  |  |  |  |  |  |  |  |  |  |
| Weight loss | 6.73 (2.07-21.92) | **0.002** | 8.30 (1.11-62.04) | **0.039** | 5.42 (1.24-23.72) | **0.025** | 2.47 (0.32-19.15) | 0.386 | 8.21 (1.94-34.82) | **0.004** |
| Appetite loss | 3.90 (1.78-8.54) | **0.001** | 2.49 (0.96-6.48) | **0.062** | 8.12 (1.86-35.49) | **0.005** | 6.53 (0.84-50.56) | **0.072** | 3.16 (1.32-7.58) | **0.010** |
| Fatigue | 3.71 (1.31-10.49) | **0.013** | 8.12 (1.08-60.84) | **0.041** | 2.20 (0.63-7.67) | **0.214** | 1.85 (0.24-14.46) | 0.557 | 4.01 (1.20-13.41) | **0.024** |
| Fever | 1.85 (0.95-3.59) | **0.070** | 1.06 (0.44-2.55) | 0.894 | 3.81 (1.24-11.67) | **0.019** | 3.03 (0.66-13.81) | **0.153** | 1.41 (0.64-3.08) | 0.394 |
| Cough | 2.85 (1.45-5.59) | **0.002** | 1.25 (0.52-3.01) | 0.613 | 8.31 (2.39-28.92) | **0.001** | 7.48 (0.97-57.95) | **0.054** | 2.03 (0.93-4.45) | **0.077** |
| Night sweats | 2.24 (0.13-4.47) | **0.021** | 1.74 (0.69-4.26) | **0.238** | 2.84 (1.00-8.05) | **0.050** | 0.81 (0.24-2.68) | 0.727 | 2.83 (1.22-6.56) | **0.015** |
| Conjunctive pallor | 3.07 (1.56-6.03) | **0.001** | 2.64 (1.05-6.62) | **0.038** | 3.44 (1.27-9.29) | **0.015** | 1.00 (0.27-3.69) | 0.999 | 4.62 (2.07-10.28) | **<0.001** |
| Karnofsky score <80% | 7.86 (3.60-17.20) | **<0.001** | 4.52 (1.74-11.75) | **0.002** | 17.55 (4.01-76.76) | **<0.001** | 4.24 (0.93-19.34) | **0.062** | 8.86 (3.54-22.19) | **<0.001** |
| WHO clinical stage |  |  |  |  |  |  |  |  |  |  |
| Stage 1-2 | 1.0 |  | 1.0 |  | 1.0 |  | 1.0 |  | 1.0 |  |
| Stage 3 | 3.21 (1.34-7.69) | **0.009** | 2.49 (0.78-7.94) | **0.123** | 3.86 (1.02-14.53) | **0.046** | 0.79 (0.18-3.52) | 0.755 | 5.07 (1.67-15.40) | **0.004** |
| Stage 4 | 7.45 (2.93-18.92) | **<0.001** | 4.74 (1.34-16.80) | **0.016** | 10.90 (2.72-43.59) | **0.001** | 2.17 (0.52-9.09) | **0.289** | 10.68 (3.13-36.49) | **<0.001** |

Data presented as n (%), or median (interquartile range).

Abbreviations: HR, hazard ratio; CI, confidence interval; BMI, body-mass index; MUAC, mid-upper arm circumference; TB, tuberculosis.

Bold p values are <0.3 indicating that the variable was included in the subsequent multivariable model.
